# Structural Design and Parameter Optimization of Magnetic Gradient Tensor Measurement System

**DOI:** 10.3390/s24134083

**Published:** 2024-06-24

**Authors:** Gaigai Liu, Yingzi Zhang, Wenyi Liu

**Affiliations:** State Key Laboratory of Dynamic Measurement Technology, North University of China, Taiyuan 030051, China; b200610@st.nuc.edu.cn (G.L.); liuwenyi@nuc.edu.cn (W.L.)

**Keywords:** magnetic anomaly detection, magnetic gradient tensor measurement, sensor array, baseline distance, fluxgate magnetometer

## Abstract

Magnetic anomaly detection (MAD) technology based on the magnetic gradient tensor (MGT) has broad application prospects in fields such as unexploded ordnance detection and mineral exploration. The difference approximation method currently employed in the MGT measurement system introduces measurement errors. Designing reasonable geometric structures and configuring optimal structural parameters can effectively reduce measurement errors. Based on research into differential MGT measurement, this paper proposes three simplified planar MGT measurement structures and provides the differential measurement matrix. The factors that affect the design of the baseline distance of the MGT measurement system are also theoretically analyzed. Then, using the magnetic dipole model, the error analysis of the MGT measurement structures is carried out. The results demonstrate that the planar cross-shaped structure is optimal, with the smallest measurement error, only 3.15 × 10^−10^ T/m. Furthermore, employing the control variable method, the impact of sensor resolution constraints, noise level, target magnetic moment, and detection distance on the design of the optimal baseline distance of the MGT measurement system is simulated and verified. The results indicate that the smaller the target magnetic moment, the farther the detection distance, the lower the magnetometer resolution, the greater the noise, and the greater the baseline distance required. These conclusions provide reference and guidance for the construction of the MGT measurement system based on triaxial magnetometers.

## 1. Introduction

The induced magnetic field generated by a ferromagnetic target is superimposed on the geomagnetic field, causing abnormal geomagnetic distribution in the space around the target. By measuring the magnetic anomaly information and performing inversion, the position and magnetic moment characteristics of the target can be determined [1,2]. Magnetic anomaly detection (MAD) technology is an excellent passive positioning technology. Due to its all-weather, low-cost, and concealment, it is widely used in military and civilian fields such as unexploded ordnance (UXO) detection [3,4,5], underwater target detection [6,7], mineral exploration [8], industrial nondestructive testing, and biomedicine [9]. Over several years of development, MAD technology has gone through three stages [10]: total magnetic intensity (TMI) detection, magnetic field vector and gradient detection, and magnetic gradient tensor (MGT) detection. Compared with TMI and magnetic field vector detection, MGT detection overcomes the influence of the geomagnetic field, provides more abundant target information, and has higher resolution [11,12]. Presently, MAD technology based on MGT has attracted widespread attention.

In recent years, numerous target inversion and positioning methods based on MGT have been proposed [13,14]. When the inversion method exhibits sufficiently high precision, the measurement of the magnetic anomaly signals becomes the primary factor affecting MAD. In practical applications, direct measurement of MGT is not feasible. Some scholars have proposed employing the difference approximation method to obtain the partial differential [15], that is, using the difference in component readings of the magnetic vector sensors to approximate the magnetic field gradient and thus obtain the MGT components. Therefore, the construction of the differential MGT measurement system using triaxial magnetometers becomes essential. Currently, the sensors that can be used to construct MGT measurement systems mainly include superconducting quantum interference devices (SQUID) and fluxgate magnetometers [16,17]. Due to its low production cost, portability, and practicality, the fluxgate magnetometer has been widely used in practice applications [18]. Although the measurement accuracy of the fluxgate magnetometer is slightly lower than that of SQUID, it can meet the needs of most target detection [19].

After the sensor type is determined, the design of the measurement structure and the selection of the baseline distance of the measurement system are necessary. Currently, the structures of MGT measurement systems constructed with fluxgate magnetometers are mainly divided into two-dimensional planar and three-dimensional spatial types [20]. For the purpose of detecting magnetic signatures of volcanoes, the United States Geological Survey (USGS) [21,22] used four triaxial fluxgate magnetometers to construct a tetrahedral structure MGT measurement system with a baseline distance of 1 m. In addition, to achieve the real-time and point-by-point detection of UXO, the Naval Surface Warfare Center Panama City Division (NSWC PCD) [23,24] developed a portable cubic structure MGT measurement system with a baseline distance of 30 cm, called Magnetic Scalar Triangulation and Ranging (Mag-STAR). This measurement structure has been adopted multiple times in subsequent studies related to the STAR method [25]. However, Xu et al. [20] have conducted research and proved that under the same measurement conditions, the measurement accuracy of the three-dimensional spatial structure is lower than that of the two-dimensional planar structure. Many subsequent studies have been based on two-dimensional planar MGT measurement structures. The United States Navy [26] constructed the first hand-held planar tensor magnetic gradiometer with a baseline distance of 0.27 m, named the room temperature Three-Sensor Gradiometer (rt-TSG). And experiments were conducted to demonstrate its real-time tracking capabilities for magnetic dipoles. However, currently, there is no consensus on the choice of planar MGT measurement structures, including planar cross-shaped or rectangular configurations [27].

Moreover, there are few relevant studies on the baseline distance of the MGT measurement system [15]. In other words, the selection basis of the baseline distances for different measurement structures is still unclear. After the measurement structure is determined, the range of the baseline distance is usually estimated based on prior information and experience. But in fact, the configuration of the baseline distance has a significant impact on the differential measurement of MGT, and it directly determines the effectiveness of the measurement. Therefore, it is necessary to conduct a study on the structure design and baseline distance selection of the MGT measurement system to find an optimal measurement structure and baseline distance, thereby improving the accuracy of tensor measurement [28].

Taking the construction of the MGT measurement system based on fluxgate magnetometers as an example, this paper designs three types of planar MGT measurement structures, namely cross-shaped, square, and triangular, and theoretically analyzes the factors that affect the selection of the baseline distance. Subsequently, the magnetic dipole model was used to simulate and analyze the structural error of the measurement system, and the optimal measurement structure was determined by comparing the measurement errors. Furthermore, the effects of sensor resolution, environmental noise, target magnetic moment, and detection distance on the baseline distance were simulated, and the criteria for determining the optimal baseline distance were given, which provided a reference for the optimal system configuration of MAD based on MGT.

## 2. Magnetic Gradient Tensor Measurement System

### 2.1. Design of Sensor Array Structure

When the detection distance is greater than 2.5 times the size of the magnetic target, the magnetic target can be regarded as a magnetic dipole [29]. According to the magnetic dipole model, the magnetic field vector $\vec{B}=\left(B_{x},B_{y},B_{z}\right)$ generated using the magnetic target can be expressed as follows:

(1) $$\vec{B}=\frac{\mu_{0}}{4\pi }\frac{3(\vec{M}\cdot \vec{r})\vec{r}-\vec{M}r^{2}}{r^{5}},$$

where $\vec{r}$ is the relative position vector from the magnetic target to the measurement point, $r=\left|\vec{r}\right|$; $\vec{M}$ is the magnetic moment vector of the magnetic target; *μ*_0_ is the magnetic permeability in vacuum, $\mu_{0}=4\pi \times 10^{-7}T\cdot m/A$.

The spatial change rate of the magnetic field vector $\vec{B}$ in the *x*, *y*, and *z* directions is called the magnetic gradient tensor [30], denoted as ***G***, and the expression is as follows:

(2) $$G=\nabla \vec{B}=\left[\begin{array}{ccc} \frac{\partial B_{x}}{\partial x} & \frac{\partial B_{x}}{\partial y} & \frac{\partial B_{x}}{\partial z}\\ \frac{\partial B_{y}}{\partial x} & \frac{\partial B_{y}}{\partial y} & \frac{\partial B_{y}}{\partial z}\\ \frac{\partial B_{z}}{\partial x} & \frac{\partial B_{z}}{\partial y} & \frac{\partial B_{z}}{\partial z} \end{array}\right]=\left[\begin{array}{ccc} B_{xx} & B_{xy} & B_{xz}\\ B_{yx} & B_{yy} & B_{yz}\\ B_{zx} & B_{zy} & B_{zz} \end{array}\right].$$

We can see that there are nine components in the MGT matrix. Essentially, the components of the MGT matrix are the first-order partial derivative of the magnetic field vector components [31]. In practical applications, it is unfeasible to measure the derivation directly. We commonly resort to finite differences for approximation. Therefore, the difference between the readings of two adjacent magnetic vector sensors is used to replace the magnetic field gradient at the center point of the two sensors to obtain the MGT. The difference approximation method is described using the following formula:

(3) $$B_{ij}=\frac{\partial B_{i}}{\partial j}\approx \frac{B_{i}^{1}-B_{i}^{2}}{d_{j}}\;(i,j=x,y,z),$$

where $B_{i}^{1}$ and $B_{i}^{2}$ are the *i* component readings of triaxial fluxgate magnetometers (TAFMs) 1 and 2, respectively; *d_j_* is the distance between these two TAFMs in the *j* direction, which is called the baseline distance.

**Figure 1:**
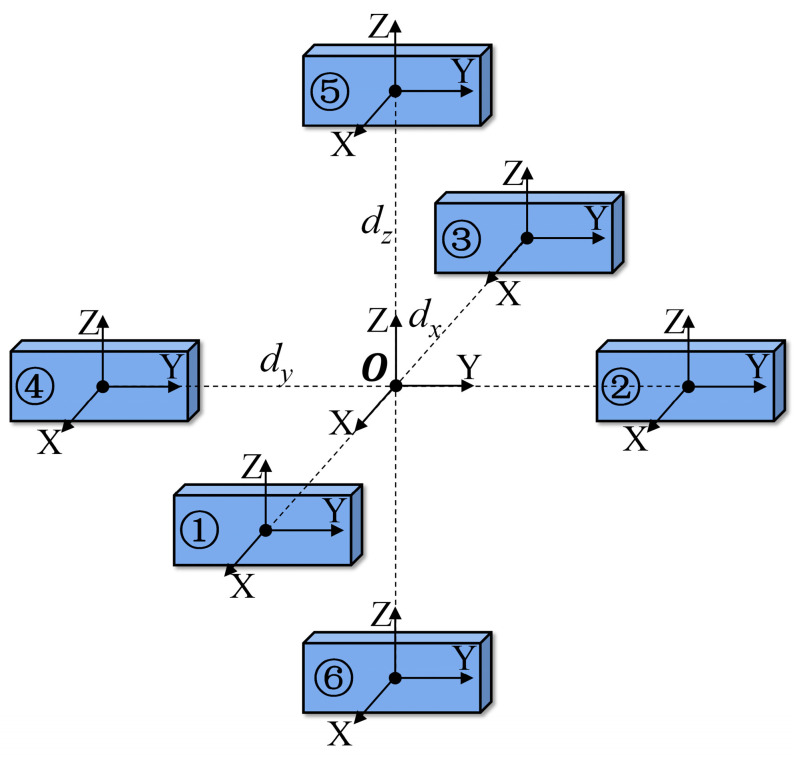
MGT measurement system composed of six three-axis fluxgate magnetometers.

Generally, using the MGT measurement system consisting of six TAFMs illustrated in Figure 1, all components of the MGT matrix can be directly obtained through the difference calculation. The calculation formula is as follows:

(4) $$G=\left[\begin{array}{ccc} (B_{x}^{1}-B_{x}^{3})/d_{x} & (B_{x}^{2}-B_{x}^{4})/d_{y} & (B_{x}^{5}-B_{x}^{6})/d_{z}\\ (B_{y}^{1}-B_{y}^{3})/d_{x} & (B_{y}^{2}-B_{y}^{4})/d_{y} & (B_{y}^{5}-B_{y}^{6})/d_{z}\\ (B_{z}^{1}-B_{z}^{3})/d_{x} & (B_{z}^{2}-B_{z}^{4})/d_{y} & (B_{z}^{5}-B_{z}^{6})/d_{z} \end{array}\right].$$

Usually, for convenience, set *d_x_* = *d_y_* = *d_z_* = *d.* However, the redundant sensors make the structure complex, and the three-dimensional design is inconvenient for installation in practical applications and can potentially introduce system errors [20].

According to Maxwell’s equations, in a passive static magnetic field, both the divergence and curl of the magnetic field vector $\vec{B}$ are equal to zero [32]:

(5) $$\left\{\begin{array}{l} div\vec{B}=\nabla \cdot \vec{B}=0\\ rot\vec{B}=\nabla \times \vec{B}=0 \end{array}\right..$$

According to Equation (5), we can derive

(6) $$\left\{\begin{array}{c} B_{xx}+B_{yy}+B_{zz}=0\\ B_{xy}=B_{yx}\\ B_{xz}=B_{zx}\\ B_{yz}=B_{zy} \end{array}\right..$$

Evidently, there are only five independent components in the MGT matrix, namely *B_xx_*, *B_xy_*, *B_xz_*, *B_yy_*, and *B_yz_*. As long as these five independent components are obtained, the MGT can be obtained. This means that the structure in Figure 1 can be optimized to reduce the number of sensors and system errors, making assembly and calibration easier.

**Figure 2:**
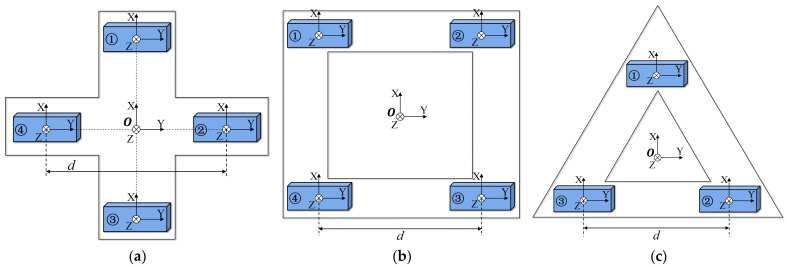
Simplified MGT measurement system. (**a**) planar cross-shaped; (**b**) square; (**c**) equilateral triangular.

By simplifying the MGT measurement structure in Figure 1, three types of planar MGT measurement structures, namely, cross-shaped, square, and equilateral triangular, are designed. The structure models are shown in Figure 2, and the corresponding differential MGT calculation formulas are described as follows:

(7) $$G_{cross-shaped}=\frac{1}{d}\left[\begin{array}{ccc} B_{x}^{1}-B_{x}^{3} & B_{x}^{2}-B_{x}^{4} & B_{z}^{1}-B_{z}^{3}\\ B_{x}^{2}-B_{x}^{4} & B_{y}^{2}-B_{y}^{4} & B_{z}^{2}-B_{z}^{4}\\ B_{z}^{1}-B_{z}^{3} & B_{z}^{2}-B_{z}^{4} & -B_{x}^{1}+B_{x}^{3}-B_{y}^{2}+B_{y}^{4} \end{array}\right],$$

(8) $$G_{square}=\frac{1}{2d}\left[\begin{array}{ccc} B_{x}^{1}+B_{x}^{2}-\left(B_{x}^{3}+B_{x}^{4}\right) & B_{x}^{2}+B_{x}^{3}-\left(B_{x}^{1}+B_{x}^{4}\right) & B_{z}^{1}+B_{z}^{2}-\left(B_{z}^{3}+B_{z}^{4}\right)\\ B_{x}^{2}+B_{x}^{3}-\left(B_{x}^{1}+B_{x}^{4}\right) & B_{y}^{2}+B_{y}^{3}-\left(B_{y}^{1}+B_{y}^{4}\right) & B_{z}^{2}+B_{z}^{3}-\left(B_{z}^{1}+B_{z}^{4}\right)\\ B_{z}^{1}+B_{z}^{2}-\left(B_{z}^{3}+B_{z}^{4}\right) & B_{z}^{2}+B_{z}^{3}-\left(B_{z}^{1}+B_{z}^{4}\right) & -B_{x}^{1}-B_{x}^{2}+B_{x}^{3}+B_{x}^{4}-B_{y}^{2}-B_{y}^{3}+B_{y}^{1}+B_{y}^{4} \end{array}\right],$$

(9) $$G_{triangular}=\frac{1}{\sqrt{3}d}\left[\begin{array}{ccc} 2B_{x}^{1}-\left(B_{x}^{2}+B_{x}^{3}\right) & 2B_{y}^{1}-\left(B_{y}^{2}+B_{y}^{3}\right) & 2B_{z}^{1}-\left(B_{z}^{2}+B_{z}^{3}\right)\\ 2B_{y}^{1}-\left(B_{y}^{2}+B_{y}^{3}\right) & \sqrt{3}\left(B_{y}^{2}-B_{y}^{3}\right) & \sqrt{3}\left(B_{z}^{2}-B_{z}^{3}\right)\\ 2B_{z}^{1}-\left(B_{z}^{2}+B_{z}^{3}\right) & \sqrt{3}\left(B_{z}^{2}-B_{z}^{3}\right) & -2B_{x}^{1}+B_{x}^{2}+B_{x}^{3}-\sqrt{3}B_{y}^{2}+\sqrt{3}B_{y}^{3} \end{array}\right].$$

### 2.2. Selection of Baseline Distance

The use of finite differences instead of partial derivatives will introduce approximation errors. Selecting an appropriate baseline distance *d* to minimize the approximation error is crucial for the accurate acquisition of the MGT. Take the measurement of *B_xx_* in Figure 1 as an example. Sensors 1 and 3 are symmetrically distributed on the *x*-axis, with the measurement point *O* (which is also the origin of the coordinate system) as the center. Here, *d* is the baseline distance of the measurement array in the *x*-direction. According to Equation (3), the measured value of *B_xx_* is $B_{xx}^{\left(m\right)}=\frac{B_{x}^{1}-B_{x}^{3}}{d}$, where $B_{x}^{1}$ and $B_{x}^{3}$ are the *x* component readings of sensors 1 and 3, respectively. The true value of *B_xx_* is $B_{xx}^{\left(t\right)}={\left.\frac{\partial B_{x}}{\partial x}\right|}_{O}$. According to the Taylor series, we can derive

(10) $$\left\{\begin{array}{l} B_{x}^{1}=B_{x}^{O}+{\left.\frac{\partial B_{x}}{\partial x}\right|}_{O}\left(x_{1}-x_{O}\right)+{\left.\frac{1}{2!}\frac{\partial^{2}B_{x}}{\partial x^{2}}\right|}_{O}{\left(x_{1}-x_{O}\right)}^{2}+{\left.\frac{1}{3!}\frac{\partial^{3}B_{x}}{\partial x^{3}}\right|}_{O}{\left(x_{1}-x_{O}\right)}^{3}+\cdots \\ B_{x}^{3}=B_{x}^{O}+{\left.\frac{\partial B_{x}}{\partial x}\right|}_{O}\left(x_{3}-x_{O}\right)+{\left.\frac{1}{2!}\frac{\partial^{2}B_{x}}{\partial x^{2}}\right|}_{O}{\left(x_{3}-x_{O}\right)}^{2}+{\left.\frac{1}{3!}\frac{\partial^{3}B_{x}}{\partial x^{3}}\right|}_{O}{\left(x_{3}-x_{O}\right)}^{3}+\cdots \end{array}\right.,$$

where $B_{x}^{O}$ is the *x*-component value of the magnetic field vector at measurement point *O*, *x_O_* is the coordinate position of the measurement point *O*, and *x*_1_ and *x*_3_ are the coordinate positions of sensor 1 and sensor 3, respectively. Given $x_{1}-x_{O}=d/2$, $x_{3}-x_{O}=-d/2$. Then, the measured value of *B_xx_* becomes

(11) $$\begin{array}{ll} B_{xx}^{\left(m\right)}=\frac{B_{x}^{1}-B_{x}^{3}}{d} & ={\left.\frac{\partial B_{x}}{\partial x}\right|}_{O}+{\left.\frac{1}{3!}\frac{\partial^{3}B_{x}}{\partial x^{3}}\right|}_{O}\frac{1}{2^{2}}d^{2}+{\left.\frac{1}{5!}\frac{\partial^{5}B_{x}}{\partial x^{5}}\right|}_{O}\frac{1}{2^{4}}d^{4}+\cdots \\ & =B_{xx}^{\left(t\right)}+\frac{1}{3!}\frac{1}{2^{2}}d^{2}{\left.\frac{\partial^{3}B_{x}}{\partial x^{3}}\right|}_{O}+{\left.\frac{1}{5!}\frac{1}{2^{4}}d^{4}\frac{\partial^{5}B_{x}}{\partial x^{5}}\right|}_{O}+\cdots \end{array}$$

It can be seen that to make $B_{xx}^{\left(m\right)}=B_{xx}^{\left(t\right)}$, a smaller baseline distance *d* is preferable. Especially when the magnetic anomaly signal is strong, and the magnetic field changes rapidly at the measurement point, the baseline distance should be further reduced to mitigate the influence of the high-order terms of the Taylor series.

However, the above conclusion is only for ideal situations. In practice, a smaller *d* is not always better.

In practical applications, the influence of noise is inevitable, mainly including sensors’ factory noise and external environmental noise. Let $S_{x}^{1}$ and $S_{x}^{3}$ represent the noise superimposed on the measurements of the *x*-component magnetic field vector at sensors 1 and 3, respectively. Through differential calculation, the error introduced by noise is

(12) $$S_{xx}=\frac{S_{x}^{1}-S_{x}^{3}}{d}.$$

Assume that $S_{x}^{1}$ and $S_{x}^{3}$ obey the normal distribution with a mean value of zero and a variance of *σ*^2^, then we can derive $S_{xx}∼(0,\frac{2\sigma^{2}}{d^{2}})$. Notably, the variance of *S_xx_* is inversely proportional to the square of the baseline distance *d* and directly proportional to the variance *σ*^2^ of the noise. Therefore, arbitrarily reducing *d* may amplify the impact of the noise. The extent of the impact depends on the strength of the magnetic anomaly signal at the measurement point. If the signal strength at the measurement point is weak, the signal may be overwhelmed by the measurement error introduced by the noise. The magnetic anomaly signal strength is closely related to the detection distance and target magnetic moment. Therefore, the selection of the baseline distance is constrained by the noise level, the magnetic moment vector of the target, and the detection distance.

Additionally, the limited resolution of the sensors that make up the MGT measurement system results in a slight measurement deviation between the sensor readings and the actual magnetic field value. This deviation is equivalent to adding noise to the measurement, which will also affect the result of the difference calculation. Let $\delta_{x}^{1}$ and $\delta_{x}^{3}$ represent the measurement deviation caused by the limited resolution of sensors 1 and 3, respectively. Then, the measurement error introduced by sensor resolution constraints is

(13) $$\delta_{xx}=\frac{\delta_{x}^{1}-\delta_{x}^{3}}{d}.$$

The lower the resolution of the sensor, the greater the measurement deviation between the sensor readings and the actual magnetic field values, and the greater the measurement error $\delta_{xx}$ introduced by the sensor resolution constraint. Therefore, to reduce the measurement error introduced by the sensor resolution, the baseline distance needs to be set larger.

In summary, the selection of the baseline distance *d* requires a careful consideration of the target magnetic moment vector, the detection distance between the measurement point and the magnetic target, the noise level, and the resolution of the sensor. A smaller baseline distance can weaken the influence of higher-order terms of the Taylor series and improve measurement accuracy. However, if *d* is too small, it may amplify the effects of noise and low sensor resolution. The theoretical measurement error introduced by the differential measurement is as follows:

(14) $$E=S_{xx}+\delta_{xx}+{\left.\frac{1}{24}\frac{\partial^{3}B_{x}}{\partial x^{3}}\right|}_{O}d^{2}+{\left.\frac{1}{1920}\frac{\partial^{5}B_{x}}{\partial x^{5}}\right|}_{O}d^{4}+\cdots .$$

## 3. Simulation

### 3.1. Determination of Optimal Array Structure

Designing a reasonable measurement structure and configuring optimal structural parameters are crucial to achieving accurate measurement of MGT. The design of the geometric structure directly affects the feasibility of the measurement. Therefore, a simulation experiment is first carried out to verify the measurement errors of MGT measurement systems with different geometric structures.

**Figure 3:**
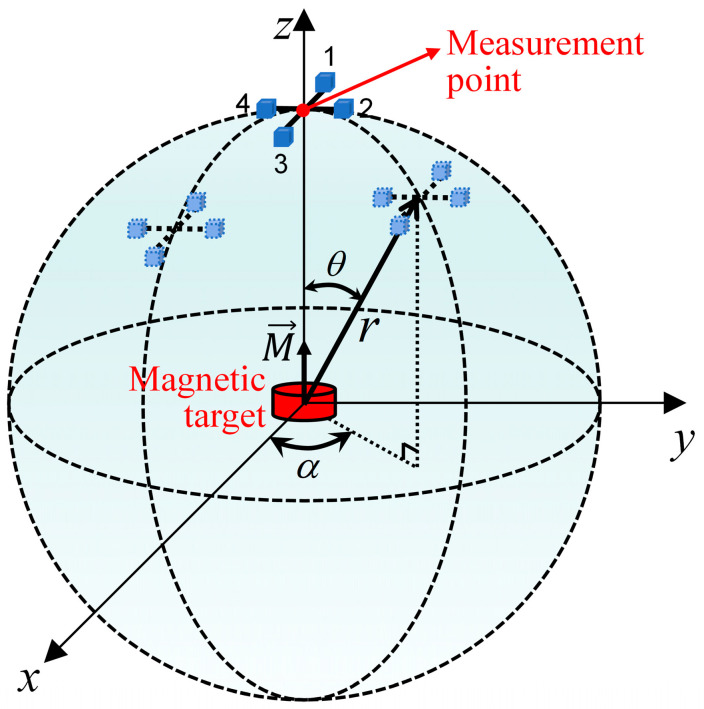
Simulation analysis model.

A Cartesian coordinate system is established with the magnetic target, having a magnetic moment vector $\vec{M}$ of (0, 0, 10,000) A·m^2^ as the origin. For convenience, most simulation experiments on MAD are performed on a single measurement point or a straight-line measurement trajectory [2]. However, the results obtained this way are one-sided and unconvincing. To evaluate the measurement error of the measurement array comprehensively, the measurement points in this paper move around the magnet target, and the motion trajectory covers the whole spherical surface, as shown in Figure 3. The radius *r* of the spherical trajectory is 10 m. *θ* and *α* are the inclination and declination of the spherical coordinate system, respectively. The baseline distance of each MGT measurement structure is set to 0.34 m.

**Figure 4:**
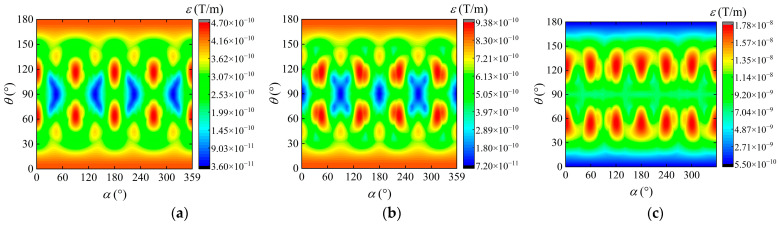
Measurement errors of different MGT measurement systems with a baseline distance of 0.34 m. (**a**) Planar cross-shaped; (**b**) square; (**c**) equilateral triangular.

Firstly, according to the magnetic dipole model, the theoretical spatial distribution of the MGT field on the spherical measurement trajectory is obtained, which is regarded as the true value of the MGT. Then, Equation (1) is used to obtain the readings from each TAFM in the measurement array, and the finite difference approximation method is employed to calculate the final measured values of the MGT matrix at each measurement point. The evaluation index *ε* for the MGT measurement error is calculated using the following formula:

(15) $$\varepsilon =\sqrt{\sum {\left(B_{ij}^{\left(m\right)}-B_{ij}^{\left(t\right)}\right)}^{2}}(i,j=x,y,z)$$

where $B_{ij}^{\left(m\right)}$ is the measured value of the MGT component, and $B_{ij}^{\left(t\right)}$ is the true value of the MGT component. The measurement errors of the planar cross-shaped, square, and equilateral triangular measurement system on the spherical measurement trajectory are shown in Figure 4.

The results indicate that under the ideal noise-free conditions, the MGT measurement system with a planar cross-shaped structure exhibits the highest measurement accuracy and smallest measurement error, followed by the square structure MGT measurement system. The maximum measurement error of the planar cross-shaped MGT measurement system on the spherical trajectory is only 4.69 × 10^−10^ T/m, which is 4.69 × 10^−10^ T/m and 1.74 × 10^−8^ T/m smaller than that of the square and equilateral triangular MGT measurement systems, respectively. The measurement error of the square structure is slightly larger than that of the cross-shaped, and its minimum measurement error is 3.60 × 10^−11^ T/m larger than that of the cross-shaped. The measurement error of the equilateral triangular MGT measurement system is the largest, and its minimum measurement error is 5.77 × 10^−10^ T/m, which is even greater than the maximum measurement error of the cross-shaped MGT measurement system. The average measurement errors of the cross-shaped, square, and equilateral triangular measurement systems are 3.15 × 10^−10^ T/m, 6.35 × 10^−10^ T/m, and 9.56 × 10^−9^ T/m, respectively. In addition, combining the structural characteristics and calculation formulas of each measurement system, it can be found that the measurement points of the planar cross-shaped and square MGT measurement structures are consistent and located exactly at the geometric center of the measurement array. In contrast, the measurement points of the equilateral triangular MGT measurement structure are inconsistent and do not coincide with the geometric center of the array. This discrepancy may be the main reason for the large measurement error of the equilateral triangular MGT measurement system. Considering the measurement error, number of sensors, and ease of installation, the planar cross-shaped structure is optimal for MGT measurement.

### 3.2. Selection of the Optimal Baseline Distance

The baseline distance plays a decisive role in the differential measurement of MGT. In this simulation, we employed the planar cross-shaped MGT measurement system and adopted the control variable method to analyze the influence of sensor resolution, environmental noise, detection distance, and target magnetic moment on the selection of the optimal baseline distance.

**Figure 5:**
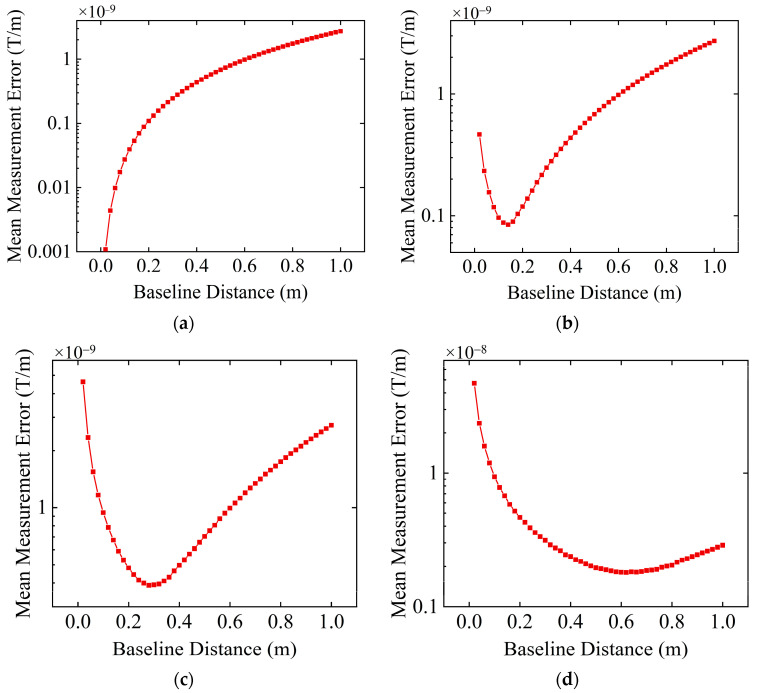
Variation in the average tensor measurement error with baseline distance under different sensor resolutions. (**a**) No sensor resolution constraints; (**b**) sensor resolution is 0.01 nT; (**c**) sensor resolution is 0.1 nT; (**d**) sensor resolution is 1 nT.

Firstly, by introducing resolution constraints to the sensors, the impact of the sensor resolution on the determination of the baseline distance was investigated. The resolution of the four fluxgate magnetometers in the cross-shaped MGT measurement system was set to 0.01 nT, 0.1 nT, and 1 nT, respectively. Other simulation conditions are consistent with Section 3.1. Under both no sensor resolution constraint and different sensor resolution constraint conditions, the variation in average MGT measurement error on the spherical trajectory with baseline distance is shown in Figure 5.

According to the theoretical analysis in Section 2.2, when there are no sensor resolution constraints and noise, only the higher-order terms of the Taylor series affect the accuracy of tensor measurement. Therefore, theoretically, the measurement error will continue to increase with an increase in baseline distance. As seen in Figure 5a, the simulation results are consistent with the theoretical analysis, further validating the correctness of the theoretical analysis. Hence, under ideal conditions without noise influence and sensor resolution constraints, to achieve more accurate tensor measurements, the baseline distance should be kept as small as possible.

From Figure 5b–d, we can find that after adding resolution constraint to the sensors, the measurement error of the MGT measurement system first decreases and then increases with increasing baseline distance. This is because when the baseline distance is too small, the readings of the magnetic field vector components from symmetrical sensors in the measurement array are very close, and the measurement deviation caused by the sensor resolution constraint has a significant impact on the results of the differential calculation, thus resulting in a relatively large measurement error. As the baseline distance increases, the influence of sensor resolution constraints gradually decreases, and therefore, the measurement error decreases. However, as the baseline distance continues to increase, the influence of higher-order terms of the Taylor series begins to become prominent and gradually dominates, and then the measurement error begins to increase. As shown in Figure 5b, when the resolution of the sensor is 0.01 nT, the average MGT measurement error on the spherical trajectory reaches the minimum value at a baseline distance of 0.14 m, which can be regarded as the optimal baseline distance. When the sensor resolution is 0.1 nT and 1 nT, the optimal baseline distances are 0.28 m and 0.62 m, respectively. At these baseline distances, the average tensor measurement errors of the cross-shaped MGT measurement system on the spherical trajectory are 8.45 × 10^−11^ T/m, 3.89 × 10^−10^ T/m, and 1.81 × 10^−9^ T/m, respectively. Comparing Figure 5b–d, we can find that as sensor resolution decreases, the optimal baseline distance of the measurement system increases. The theoretical analysis in Section 2.2 indicates that the lower the sensor resolution, the larger the required baseline distance. Therefore, the theoretical analysis is consistent with the simulation results.

**Figure 6:**
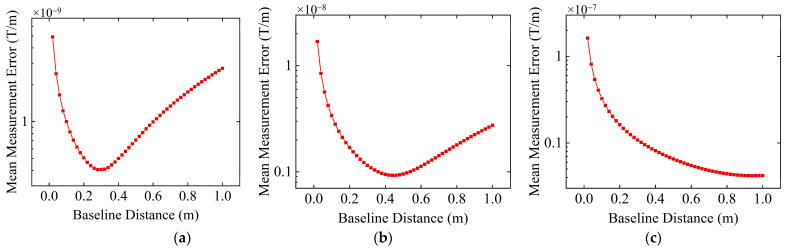
Variation in the average tensor measurement error with baseline distance after adding different levels of noise, with the sensor resolution controlled at 0.1 nT. (**a**) Standard deviation σ of the noise is 0.01 nT; (**b**) standard deviation σ of the noise is 0.1 nT; (**c**) standard deviation σ of the noise is 1 nT.

In actual measurements, the influence of environmental noise is inevitable. Assume that the resolution of the sensor is 0.1 nT, and Gaussian white noise with a mean of zero and a standard deviation of σ is added to the measurement values of the sensors as environmental noise. Other simulation conditions remain consistent with Section 3.1. The values of σ are set to 0.01 nT, 0.1 nT, and 1 nT, and the variation in the average tensor measurement error on the spherical trajectory with baseline distance under the influence of different noise levels is illustrated in Figure 6.

From Figure 6, we can find that after adding noise, the measurement error of the MGT measurement system also initially decreases and then increases as the baseline distance increases. When the baseline distance is small, noise and sensor resolution constraints have a greater impact on the measurement, and the real signal is almost drowned, thus resulting in a relatively large measurement error. As the baseline distance gradually increases, the influence of noise and sensor resolution constraints gradually decreases, and the measurement error also decreases. However, as the baseline distance continues to increase, the influence of high-order terms in the Taylor series becomes prominent, and measurement errors caused by high-order terms of the Taylor series gradually increase. Compared with the measurement error without noise influence in Figure 5c, the maximum measurement error at a noise level of 0.01 nT in Figure 6a increases by 2.80 × 10^−10^ T/m, and the minimum measurement error increases by 1.63 × 10^−11^ T/m when the baseline distance changes from 0.02 m to 1 m. When the noise level is 0.01 nT, 0.1 nT, and 1 nT, the corresponding optimal baseline distances are 0.28 m, 0.44 m, and 0.94 m, respectively. At these baseline distances, noise and sensor resolution constraints have less impact on the differential calculation, and the measurement error caused by higher-order terms of the Taylor series is also not obvious. Thus, the tensor measurement error reaches a minimum. Comparing Figure 6a–c, it can be seen that as the noise level increases, the optimal baseline distance of the MGT measurement system increases accordingly, which is consistent with the theoretical analysis results in Section 2.2, that is, the greater the noise, the larger the baseline distance needs to be set.

**Figure 7:**
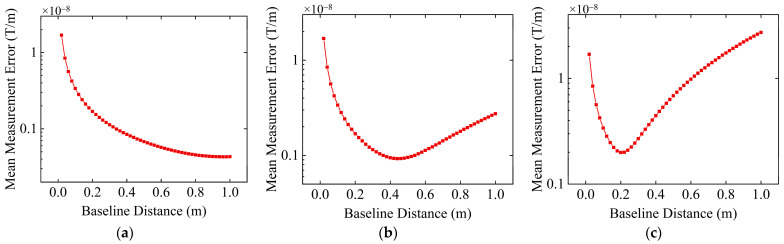
Variation in the average tensor measurement error with baseline distance under different target magnetic moments, with the sensor resolution controlled at 0.1 nT and environmental noise level σ controlled at 0.1 nT. (**a**) Target magnetic moment *M* is 1000 A·m^2^; (**b**) target magnetic moment *M* is 10,000 A·m^2^; (**c**) target magnetic moment *M* is 100,000 A·m^2^.

Moreover, assuming that the resolution of the sensors is 0.1 nT and the level σ of the environmental noise is 0.1 nT, the variation in the average MGT measurement error on the spherical trajectory with the baseline distance is studied when the target magnetic moments are 1000 A·m^2^, 10,000 A·m^2^, and 100,000 A·m^2^, and the results are shown in Figure 7. Other simulation parameters remain consistent with previous simulation experiments.

When the detection distance is 10 m and the target magnetic moments are 1000 A·m^2^, 10,000 A·m^2^, and 100,000 A·m^2^, the optimal baseline distances of the MGT measurement system are 0.96 m, 0.44 m, and 0.20 m, respectively. That is, under the same sensor resolution and environmental noise conditions, when the detection distance is constant, the larger the target magnetic moment, the smaller the optimal baseline distance. This is because the larger the target magnetic moment, the stronger the magnetic anomaly signal intensity, and the greater the gradient or change rate of the magnetic anomaly signal at the measurement point. Therefore, the baseline distance should be smaller to reduce the influence of higher-order terms in the Taylor series. However, the reduction in baseline distance amplifies the effects of environmental noise and sensor resolution. Despite this, due to the strong magnetic anomaly signal, the signal-to-noise ratio is still high, and the influence of the environmental noise and sensor resolution on the difference calculation results is slighter than the influence of higher-order terms in the Taylor series. Until the baseline distance becomes small enough so that the impact of higher-order terms is small, and at the same time, the influence of noise and sensor resolution constraints is not significant, the measurement errors caused by the three factors reach a tipping point (which is a minimum value), and the optimal baseline distance appears. If the baseline distance continues to decrease, the effects of noise and sensor resolution will be so great that measurement errors begin to increase. Therefore, the essence of finding the optimal baseline distance is to find a balance point between the effects of higher-order terms, environmental noise, and sensor resolution.

Since some studies [33] have shown that changes in the direction of the target magnetic moment have an insignificant impact on the measurement error of the MGT system, we have not discussed it here.

**Figure 8:**
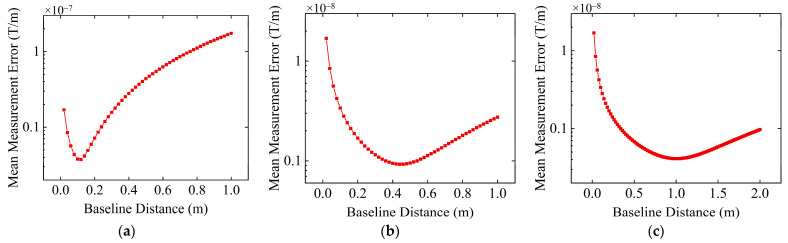
Variation in the average tensor measurement error with baseline distance under different detection distances, with sensor resolution controlled at 0.1 nT, environmental noise level σ controlled at 0.1 nT, and target magnetic moment *M* controlled at 10,000 A·m^2^. (**a**) Detection distance *r* is 5 m; (**b**) detection distance *r* is 10 m; (**c**) detection distance *r* is 15 m.

In addition, when the target magnetic moment is fixed at 10,000 A·m^2^, the sensor resolution is 0.1 nT, and the environmental noise level is 0.1 nT, the variation of the average tensor measurement error on the spherical trajectory with the baseline distance is studied under the conditions of detection distances of 5 m, 10 m, and 15 m. The results are shown in Figure 8.

When the detection distances are 5 m, 10 m, and 15 m, the optimal baseline distances are 0.12 m, 0.44 m, and 1.02 m, respectively, and the average tensor measurement errors under the optimal baseline distance conditions are 3.73 × 10^−9^ T/m, 9.25 × 10^−10^ T/m, 4.11 × 10^−10^ T/m. This result indicates that when the target magnetic moment is constant, the farther the detection distance, the larger the optimal baseline distance of the MGT measurement system. This is because when the detection distance is far, the magnetic anomaly signal is weak, resulting in a low signal-to-noise ratio. Therefore, the baseline distance needs to be relatively large to reduce the influence of noise and sensor resolution constraints on the measurement, although the increase in baseline distance will increase the impact of the high-order terms in the Taylor series. The optimal baseline distance is found when the measurement errors caused by noise and sensor resolution become relatively small, and the measurement errors caused by higher-order terms of the Taylor series begin to increase significantly, resulting in the total measurement error caused by these three factors reaching a minimum value. On the contrary, when the detection distance is close, a smaller baseline distance is required to achieve a relatively small measurement error.

When the environmental noise and sensor resolution are constant, an increase in the detection distance at a certain target magnetic moment is equivalent to a decrease in the target magnetic moment at a certain detection distance. Therefore, the changes observed in Figure 8a–c are equivalent to the simulation results in the above simulation experiment, where the detection distance is constant and the target magnetic moment decreases.

## 4. Conclusions

The research on MAD based on MGT mainly focuses on inversion methods, ignoring the importance of accurate tensor measurements. The currently used MGT measurement method, namely, finite differences instead of partial derivatives, introduces measurement errors. Designing a reasonable geometric structure and configuring accurate structural parameters are crucial to reducing measurement errors and achieving accurate measurement of target MGT information.

The design of the measurement array structure determines the feasibility of measurement. In this paper, tensor measurement theory is introduced, and three planar MGT measurement structures are proposed. By comparing the measurement errors of the planar structures, the optimal measurement structure is determined through simulation using the magnetic dipole model.

The baseline distance determines the effectiveness of the difference approximation. This paper theoretically analyzes factors that may affect the selection of the baseline distance and conducts simulation experiments based on the optimal measurement structure proposed above to verify the theoretical analysis results. The simulation results align with the theoretical analyses. Both show that the determination of the baseline distance is related to the magnetic anomaly signal strength at the measurement point (which is equivalent to the target magnetic moment and detection distance), environmental noise level, and sensor resolution. The smaller the target magnetic moment, the farther the detection distance, the lower the resolution of the magnetometer, and the higher the environmental noise, the larger the baseline distance needs to be set, and vice versa. These results provide a reference for the design and parameter optimization of the MGT measurement system. However, only simulation verification is performed in this paper. For practical applications, further research is required. In the future, we will focus on developing experimental systems and environments, exploring experimental verification methods, and further verifying the feasibility of the proposed method in practical application scenarios.

## Data Availability

The original contributions presented in the study are included in the article, further inquiries can be directed to the corresponding author.
